# Systematic review and meta-analysis of the efficacy of serum neuron-specific enolase for early small cell lung cancer screening

**DOI:** 10.18632/oncotarget.17825

**Published:** 2017-05-11

**Authors:** Lang Huang, Jian-Guo Zhou, Wen-Xiu Yao, Xu Tian, Shui-Ping Lv, Ting-You Zhang, Shu-Han Jin, Yu-Ju Bai, Hu Ma

**Affiliations:** ^1^ Department of Oncology, Affiliated Hospital of Zunyi Medical University, Zunyi 563000, China; ^2^ Department of Oncology, Affiliated Cancer Hospital of Medical School, University of Electronic Science and Technology of China, Sichuan Cancer Hospital and Institute & Cancer, The Second People's Hospital of Sichuan Province, Chengdu 610000, China; ^3^ Chongqing Cancer Hospital and Institute, Chongqing 40030, China; ^4^ Department of Cardiology and Endodontics, Affiliated Stomatological Hospital of Zunyi Medical University, Zunyi 563000, China

**Keywords:** neuron-specific enolase, diagnosis accuracy, small cell lung cancer, systematic review, meta-analysis

## Abstract

We performed a pooled analysis of the efficacy of serum neuron-specific enolase (NSE) levels for early detection of small cell lung cancer (SCLC) in patients with benign lung diseases and healthy individuals. Comprehensive searches of several databases through September 2016 were conducted. The quality of the included studies was assessed using the Quality Assessment of Diagnostic Accuracy Studies (QUADAS-2) tool. Ultimately, 33 studies containing 9546 samples were included in the review. Pooled sensitivity of NSE for detecting SCLC was 0.688 (95%CI: 0.627-0.743), specificity was 0.921 (95%CI: 0.890-0.944), positive likelihood ratio was 8.744 (95%CI: 6.308-12.121), negative likelihood ratio was 0.339 (95%CI: 0.283- 0.405), diagnostic odds ratio was 25.827 (95%CI: 17.490- 38.136) and area under the curve was 0.88 (95%CI: 0.85- 0.91). Meta-regression indicated that study region was a source of heterogeneity in the sensitivity and joint models, while cut-off level was a source in the joint model. Subgroup analysis showed that enzyme linked immunosorbent assays had the highest sensitivity and radioimmunoassay assays had the highest specificity. The diagnostic performance was better in Europe [sensitivity: 0.740 (95%CI: 0.676-0.795), specificity: 0.932 (95%CI: 0.904-0.953)] than in Asia [sensitivity: 0.590 (95%CI: 0.496- 0.678), specificity: 0.901 (95%CI: 0.819-0.948)]. In Europe, 25 ng/ml is likely the most suitable NSE cut-off level. NSE thus has high diagnostic efficacy when screening for SCLC, though the efficacy differs depending on study region, assay method and cut-off level. In the clinic, NSE measurements should be considered along with clinical symptoms, image results and histopathology.

## INTRODUCTION

Lung cancer is the leading cause of cancer death in China and worldwide for both men and women. Small cell lung cancer (SCLC) accounts for approximately 13%-15% of lung cancer cases [1, 2]. SCLC is an aggressive neuroendocrine tumor with clinical and pathological characteristics distinct from other histological types. Its 5-year overall survival rate is a mere 6.3%, and there has been little progress in several decades [3]. Moreover, for advanced stage SCLC, the median survival time is only about 9-10 months [4, 5]. Clearly, therefore, only early diagnosis with timely appropriate treatment has the potential to provide a more favorable outcome for SCLC patients.

Neuron-specific enolase (NSE) is a glycolytic neurospecific isozyme of enolase [6]. This enzyme is a well-established marker whose serum levels are used to support an initial diagnosis of SCLC [7]. Several studies have shown that NSE has a high diagnostic capacity for SCLC patients [8–10]. Likewise, a meta-analysis [11] showed that NSE has a high index for diagnosis of SCLC. It is therefore recommended by the European Group on Tumor Markers guidelines that NSE be used for differential diagnosis in patients with lung tumors of unknown origin.

At present, enzyme linked immunosorbent assays (ELISA), electro-chemiluminescence immunoassays (ECSIA) and radioimmunoassay assays (RIA) are all used to determine serum NSE levels. This raises uncertainty as to whether the diagnostic efficacy of NSE may differ among the various detecting methods. In addition, there is also uncertainty as to whether tumor location influences the sensitivity and specificity of NSE. Finally the reported cut-off levels vary, so an optimal clinical threshold level for NSE needs to be determined. We therefore conducted a systematic review and meta-analysis to assess the efficacy of serum NSE levels for early detection of SCLC in patients with benign lung diseases and healthy individuals.

## RESULTS

### Literature research and characteristics of studies

**Figure 1:**
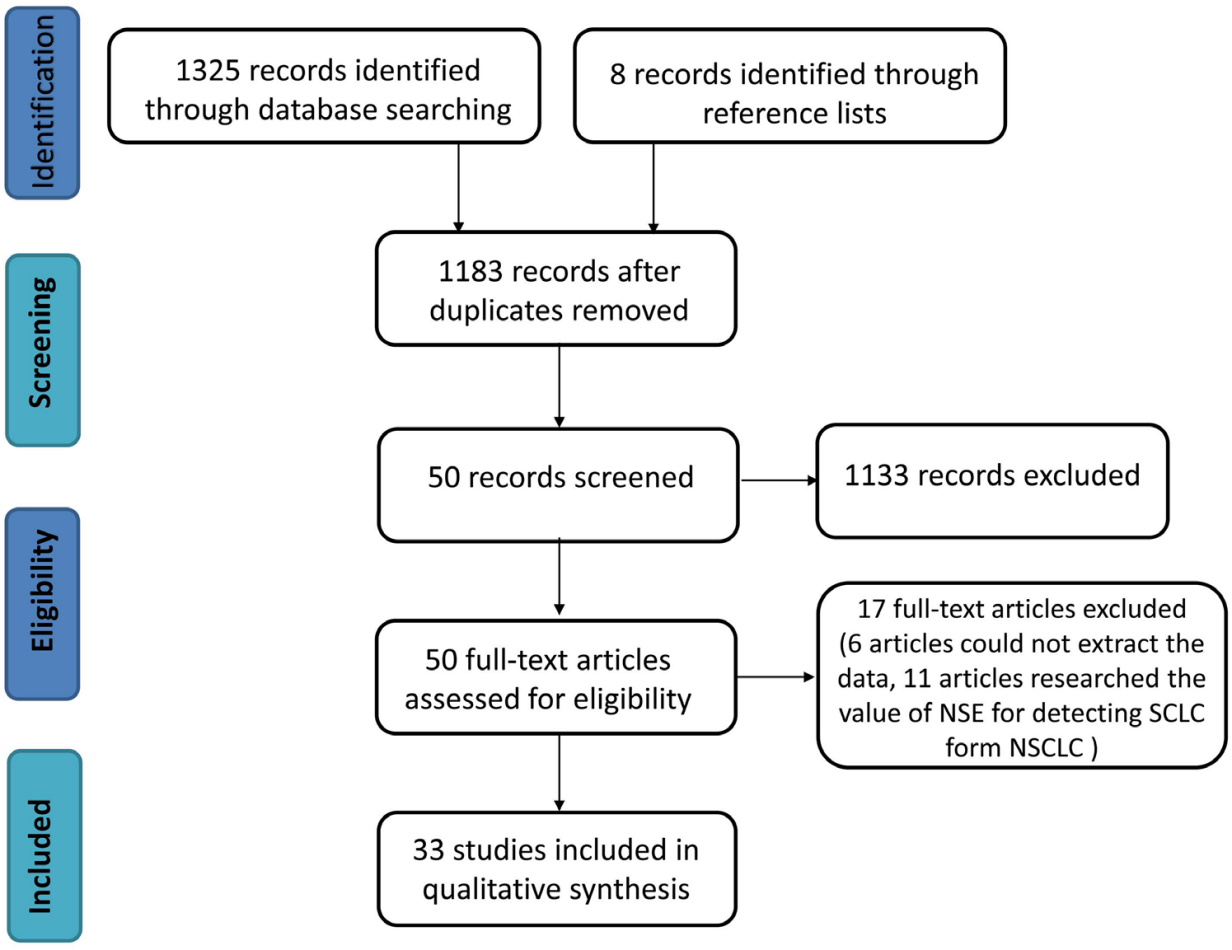
Flow chart of the systematic review process

As showed in Figure 1, 1325 literature citations were identified from database searches, and 8 citations were identified from reference lists. Ultimately, 33 studies [8–10, 12–41] met the inclusion criteria and were included in our review. Among the 9546 samples studied, 2990 were diagnosed as SCLC.

All studies were published between 1985 and 2013, Body *et al*. [14] and STIEBER *et al*. [25] each had two different NSE cut-off levels for detecting SCLC. Nineteen studies were from Europe, and 14 were form Asia. The NSE cut-off levels reported in those studies ranged from 7.5 ng/ml to 35 ng/ml. No NSE cut-off level was reported in two studies [40, 41]. Three different methods were used to detect NSE: ELISA was available in 15 trials with 3498 samples; 14 trials with 3838 samples used RIA; and 6 trials with 2210 samples used ECISA. The characteristics of the included studies are shown in Table 1.

**Table 1:** The characteristics of the included studies

Study	Year	Country	TP	FP	FN	TN	Detection Method	Cut-off (ng/ml)
Body(a)	1992	Belgium	79	6	18	94	RIA	11.7
Body(b)	1992	Belgium	86	11	11	89	RIA	9.2
Burghuber	1990	Austria	63	53	18	299	RIA	12.3
Dienemann	1994	Germany	42	26	13	163	ELISA	13.7
Dilmaghani-Marand	2013	Germany	50	0	0	90	ELISA	29.5
Ebert	1996	Germany	95	34	35	348	RIA	13.8
ESSCHER	1985	Sweden	74	4	29	368	RIA	25
Feng	2010	China	4	72	4	192	ECSIA	15.2
FRANJEBIC	2012	Croatia	201	10	127	195	ECSIA	NA
Gruber	2008	Germany	78	2	116	316	RIA	35
Han	1994	China	10	14	8	31	ELISA	20
HOLDENRIEDER	2010	Germany	44	2	9	38	ECSIA	NA
Jaques	1993	Japan	75	0	146	87	RIA	25
Keller	1998	Germany	52	50	8	302	ELISA	18
Lamy	2000	France	110	4	36	55	ELISA	17
Li	2003	China	18	5	12	55	ELISA	8
Molina	2008	Spain	78	32	18	385	ELISA	25
Molina	2009	Spain	114	50	61	577	ELISA	25
Muley	2003	Germany	138	71	50	744	ECSIA	21.6
Niklinski	1993	Poland	30	0	18	15	ELISA	15
NISMAN	2009	Japan	18	15	19	110	ELISA	22
Pan	2002	China	19	51	5	99	ELISA	13
Pinson	1997	Belgium	47	20	17	96	RIA	12.5
Poposka	2004	Macedonia	24	11	9	79	RIA	16.6
Scagliotti	1989	Italy	44	5	18	42	RIA	12
Shibayama	2001	Japan	49	3	65	103	ELISA	7.5
Stieber	1993	Germany	34	14	28	259	RIA	18
STIEBER(a)	1999	Japan	39	4	48	70	RIA	11.9
STIEBER(b)	1999	Japan	25	1	61	73	RIA	23.1
Takada	1996	Japan	73	6	28	108	ELISA	10.6
Yang	2000	China	18	5	12	55	ELISA	8
Yang(a)	2005	China	14	41	7	103	ECSIA	16.3
Yang(b)	2005	China	40	16	23	65	ECSIA	16.3
Zhang	2002	China	6	22	2	106	RIA	20
Zhou	1995	China	16	13	4	72	ELISA	20.8

ELISA: enzyme linked immunosorbent assay; ECSIA: electro-chemiluminescence; Immunoassay assay; RIA= radioimmunoassay assay; TP=true positive; FP: false positive; FN: false negative; TN=true negative.

### Quality assessment

**Figure 2:**
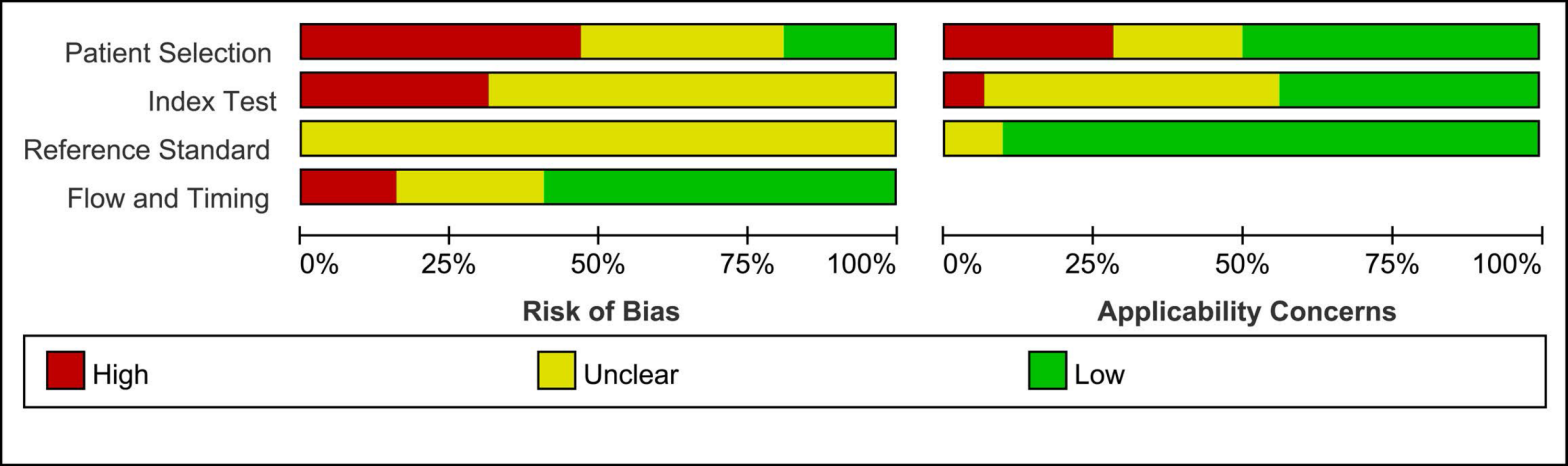
Risk of bias and applicability concerns summary

**Figure 3:**
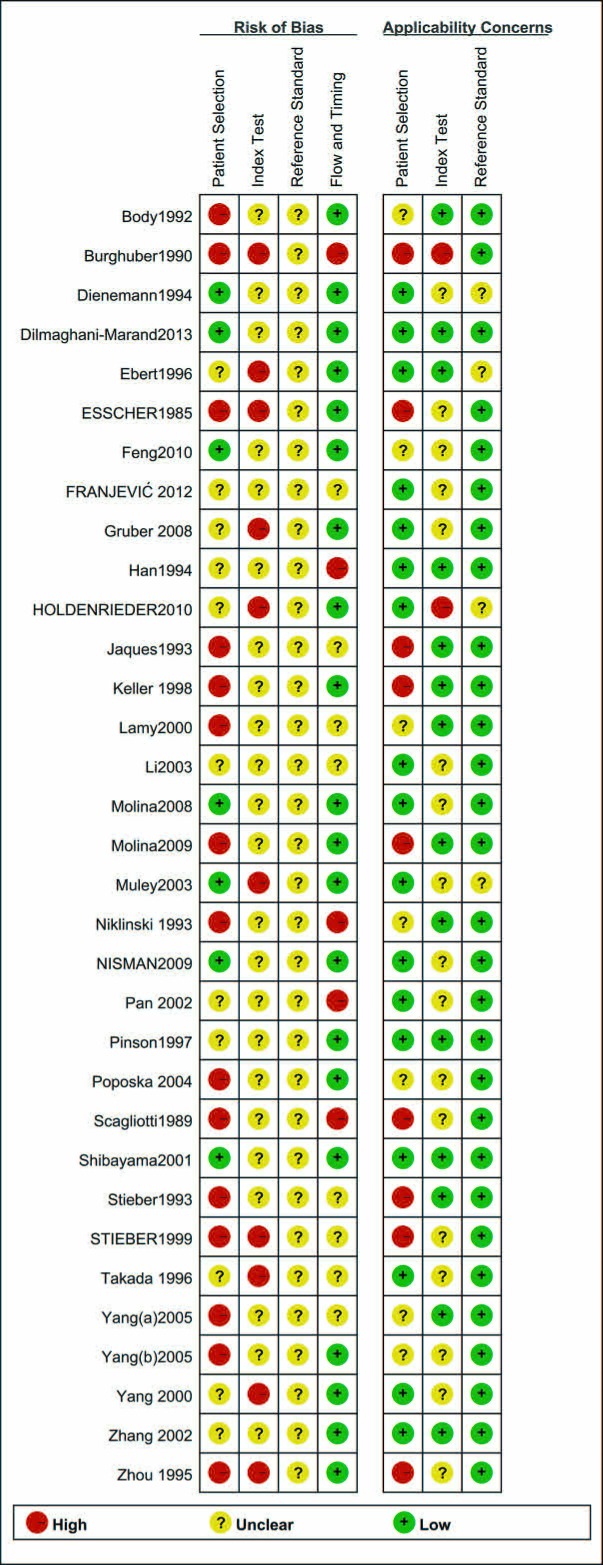
Risk of bias and applicability concerns graph

Quality Assessment of Diagnostic Accuracy Studies 2 (QUADAS-2) tool was used to assess the methodological quality of included studies. Patient selection showed high bias in 15 studies. Ten studies were designated as having unclear bias in their index tests, and 19 studies were allocated as low bias in their flow and timing. Regarding applicability concerns, 9 studies showed high bias in patient selection, 2 studies had applicability concerns as high bias, and 30 studies were allocated as low bias in reference standard. As shown in Figures 2 and Figure 3, some studies were rated as high risk, and the item flow and timing for risk of bias may have impacted the pooled effects (Supplementary Data 2).

### Diagnostic performance

**Figure 4:**
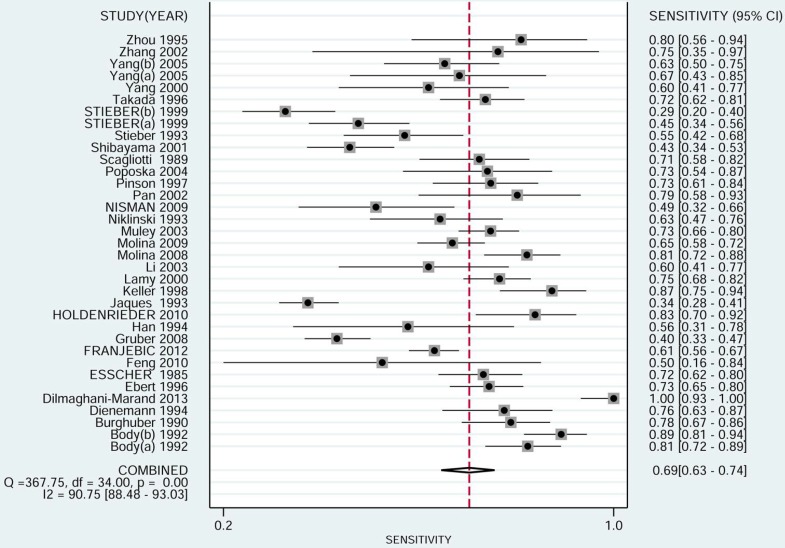
Forest plot estimating the sensitivity of NSE in SCLC patients in the selected studies (Point estimates for sensitivity and 95% CIs are shown with pooled estimates; NSE = neuron-specific enolase; SCLC = small cell lung cancer; CI = confidence interval; Q = Cochran Q statistic).

**Figure 5:**
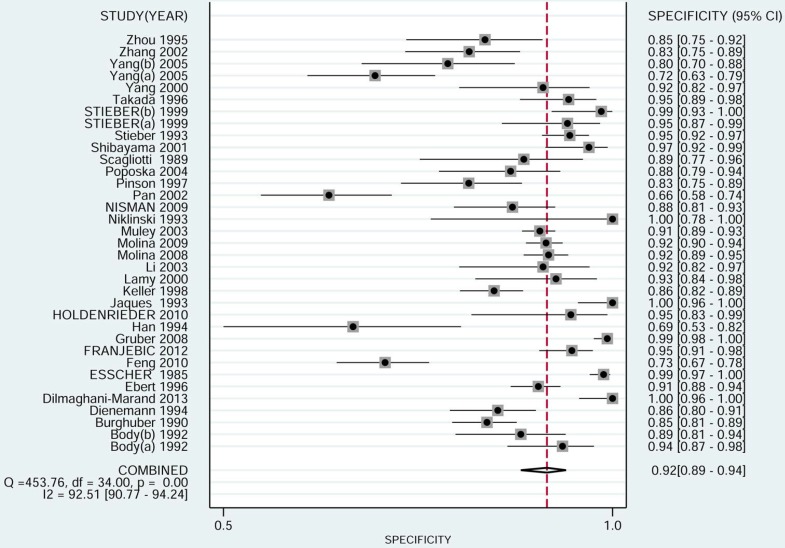
Forest plot estimating the specificity of NSE in SCLC patients in the selected studies (Point estimates for specificity and 95% CIs are shown along with pooled estimates; NSE = neuron-specific enolase; SCLC = small cell lung cancer; CI = confidence interval; Q = Cochran Q statistic).

**Figure 6:**
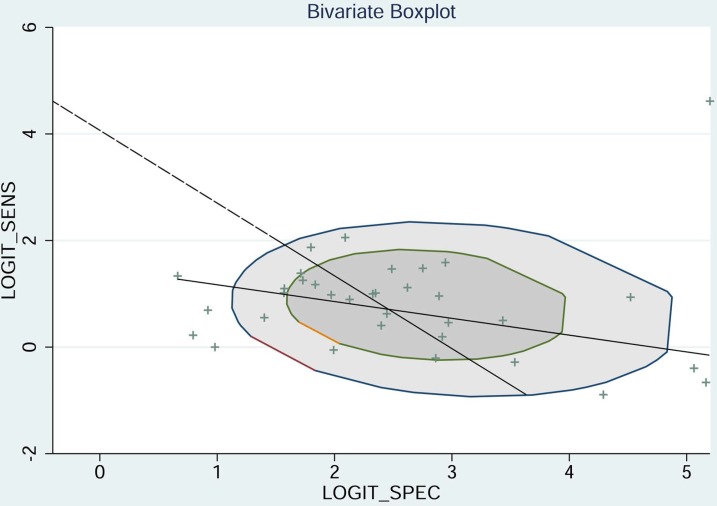
Bivariate boxplot of sensitivity and specificity in the 33 included trials

**Figure 7:**
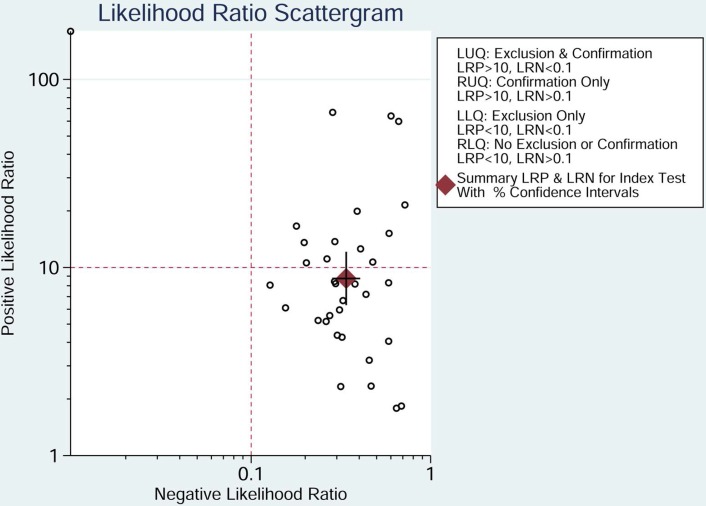
Likelihood ratio scattergram evaluating the positive likelihood ratios of NSE in the diagnosis of SCLC (Point estimates for positive likelihood ratio and 95% CIs are shown along with pooled estimates; NSE = neuron-specific enolase; SCLC = small cell lung cancer).

**Figure 8:**
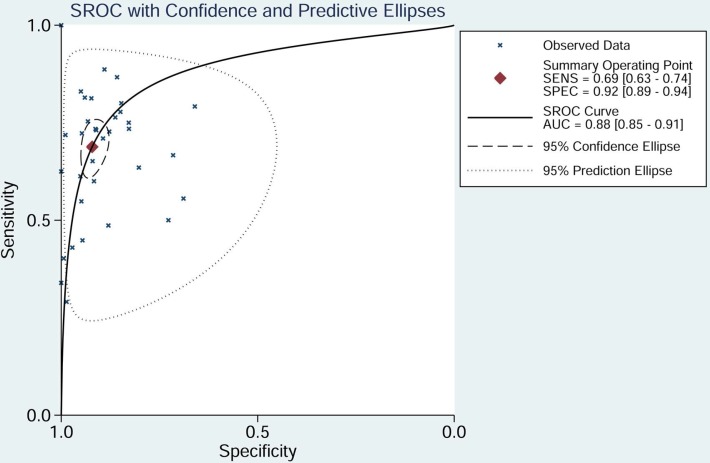
SROC curve for NSE in the diagnosis of SCLC (AUC = area under the curve; NSE = neuron-specific enolase; SCLC = small cell lung cancer; SROC = summary receiver-operating characteristic).

**Figure 9:**
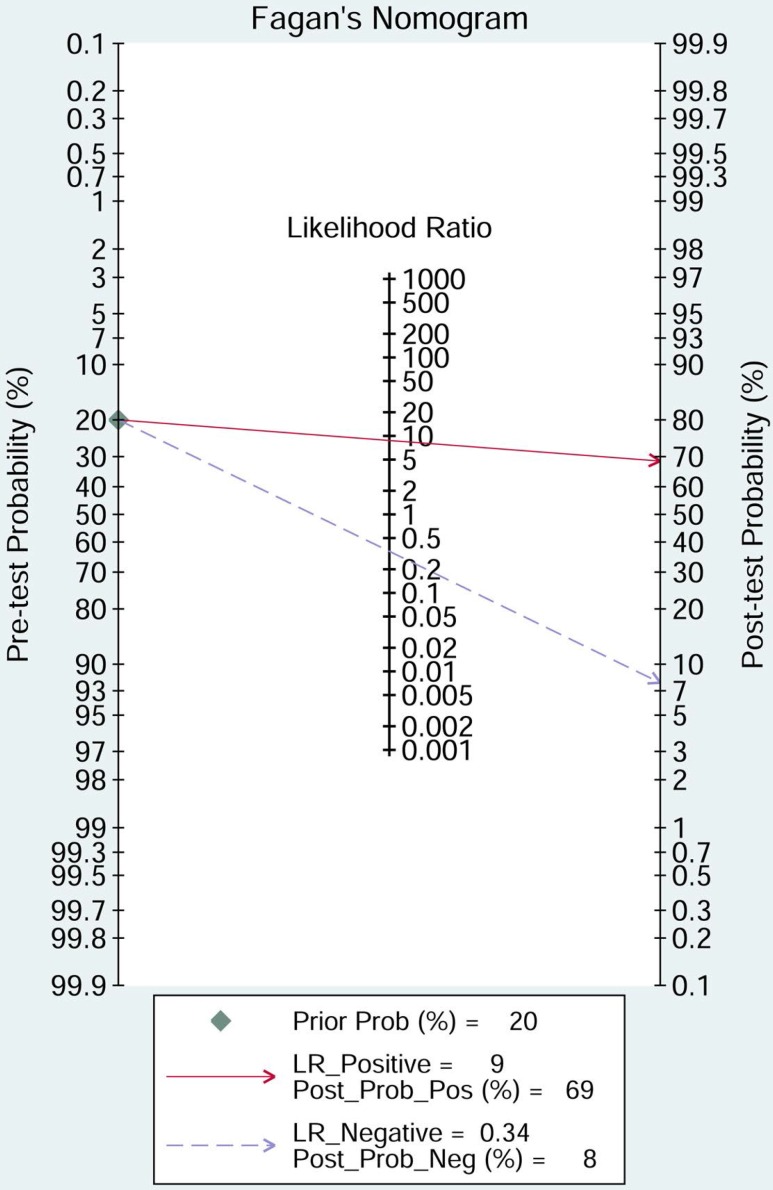
Fagan diagram evaluating the overall value of SCLC for the diagnosis of SCLC (NSE = neuron-specific enolase; SCLC = small cell lung cancer).

The pooled sensitivity of NSE for detecting SCLC was 0.688 (95%CI: 0.627-0.743) (Figure 4), the specificity was 0.921(95%CI: 0.890-0.944) (Figure 5), the positive likelihood ratio (PLR) was 8.744 (6.308-12.121), the negative likelihood ratio (NLR) was 0.339 (95%CI: 0.283, 0.405), and diagnostic odds ratio (DOR) was 25.827 (95%CI: 17.490- 38.136). A bivariate boxplot (Figure 6) showed that significant heterogeneity was present in our review. The summary LRP and LRN for NSE was on the right, under quadrant (LUQ) (Figure 7), and the exclusion and confirmation of NSE for detecting SCLC was not bad. The summary receiver operator characteristic (SROC) curve area under the curve (AUC) was 0.88 (95%CI: 0.85-0.91), and high diagnostic performance was indicated (Figure 8). The clinical utility of NSE for early SCLC screening was good, and Fagan's nomogram (Figure 9) showed that the post-test probability (PLR: 70%, NLR: 7%) differed substantially from the pretest probability (20%).

### Meta-regression analysis

Because the *I*^2^ of 99.25 (95%CI: 98.91-99.59) and boxplot (Figure 6) showed that heterogeneity existed in our review, meta-regression analysis was conducted to investigate potential sources of heterogeneity. Detection method, study region, cut-off level and sample size (n ≥ 150 vs n < 150) were included in the meta-regression analysis of sensitivity, specificity and joint models. The results (Table 2) indicated that region may be the source of the heterogeneity in the sensitivity and joint models, while cut-off value was a likely source in the joint model.

**Table 2:** The result of meta-regression

**Sensitivity**				
**Parameter**	**Estimate(95%CI)**	**Coef**	**Z**	***P*>|z|**
Detection method	0.68 [0.62 - 0.74]	0.76	−0.52	0.60
Region	0.58 [0.49 - 0.67]	0.34	−2.82	0.00*
Cut-off	0.68 [0.62 - 0.74]	0.77	−0.05	0.96
Sample size^#^	0.71 [0.62 - 0.78]	0.87	0.65	0.51
**Specificity**				
**Parameter**	**Estimate(95%CI)**	**Coef**	**Z**	***P*>|z|**
Detection method	0.92 [0.89 - 0.94]	2.44	−0.29	0.77
Region	0.89 [0.83 - 0.94]	2.13	−1.40	0.16
Cut-off	0.92 [0.89 - 0.95]	2.50	0.30	0.77
Sample size^#^	0.90 [0.84 - 0.93]	2.15	−1.75	0.08
**Joint model**				
**Parameter**	**I-squared(95%CI)**	**LRTChi**	***P* Value**	
Detection method	0.00 [0.00 - 100.00]	0.82	0.66	
Region	85.04 [68.84- 100.00]	13.37	0.00*	
Cut-off	94.18 [89.24 - 99.13]	34.38	0.00*	
Sample size^#^	32.77 [0.00 - 100.00]	2.97	0.23	

^#Total patient <150 cases or ≥150 cases;* P<0.05^.

### Subgroup analysis

The results of subgroup analysis of the different regions and detection methods are shown in Table 3. For regions, the sensitivity and specificity were 0.740 (95%CI: 0.676-0.795) and 0.932 (95%CI: 0.904-0.953) in Europe, which were much higher than in Asia [sensitivity=0.590 (95%CI: 0.496- 0.678); specificity=0.901 (95%CI: 0.819-0.948)]. Regarding detection methods, when ELISA was used to detect NSE, the sensitivity was 0.722 (95%CI: 0.623-0.803) and specificity was 0.910 (95%CI: 0.858-0.944); when RIA was used, the sensitivity was 0.655 (95%CI: 0.545-0.751) and specificity was 0.949 (95%CI: 0.904-0.973); when ECISA was used, the sensitivity was 0.674 (95%CI: 0.584-0.753) and specificity was 0.869 (95%CI: 0.766-0.931). In Europe, ELISA had the highest sensitivity (0.792, 95%CI: 0.617- 0.900), while RIA had the highest specificity (0.936, 95%CI: 0.882-0.966). Likewise, in Asia, the highest sensitivity (0.410, 95%CI: 0.523- 0.729) was obtained when ELISA was used for detection, and the highest specificity (0.970, 95%CI: 0.870- 0.994) was obtained when RIA was used.

**Table 3:** Subgroup analysis of study region and detection method

Subgroup		No. of Trials	No. of Patients	Sensitivity	Specificity
Region	Europe	21	7243	0.740 (95%CI: 0.676- 0.795)	0.932 (95%CI: 0.904-0.953)
	Asia	14	2303	0.590 (95%CI: 0.496- 0.678)	0.901 (95%CI: 0.819-0.948)
Detection method	ELISA	15	3498	0.722 (95%CI: 0.623- 0.803)	0.910 (95%CI: 0.858- 0.944)
	RIA	14	3838	0.655 (95%CI: 0.545- 0.751)	0.949 (95%CI: 0.904- 0.973)
	ECISA	6	2210	0.674 (95%CI: 0.584- 0.753)	0.869 (95%CI: 0.766- 0.931)
Europe	ELISA	8	2541	0.792 (95%CI: 0.617- 0.900)	0.922 (95%CI: 0.866-0.955)
	RIA	10	3073	0.720 (95%CI: 0.632- 0.794)	0.936 (95%CI: 0.882-0.966)
	ECISA	3	1629	0.673(95%CI: 0.633- 0.712)	0.922 (95%CI: 0.904-0.937)
Asia	ELISA	7	957	0.653 (95%CI: 0.543- 0.750)	0.886 (95%CI: 0.783- 0.944)
	RIA	4	765	0.655 (95%CI: 0.299- 0.532)	0.970 (95%CI: 0.870- 0.994)
	ECISA	3	581	0.630 (95%CI: 0.523- 0.729)	0.736 (95%CI: 0.695- 0.775)

ELISA: enzyme linked immunosorbent assay; ECSIA: electro- chemiluminescence immunoassay; RIA= radioimmunoassay assay.

Different NSE cut-off levels were also analyzed (Table 4), and different sensitivities and specificities were found. Taking all countries into consideration, the highest sensitivity (0.733, 95%CI: 0.416-0.914) and specificity (0.986, 95%CI: 0.943-0.997) were found when the NSE cut-off level was 25 ng/ml; when cut-off level was 12.5 ng/ml, the sensitivity (0.684, 95%CI: 0.605- 0.754) and specificity (0.919, 95%CI: 0.689- 0.951) were the lowest. Consistent with the values of all countries, in Europe the sensitivity (0.803, 95%CI: 0.460-0.951) and specificity (0.983, 95%CI: 0.924-0.996) were highest when the NSE cut-off level was 25 ng/ml. The sensitivity (0.723, 95%CI: 0.636-0.796) and specificity (0.940, 95%CI: 0.900- 0.964) were lowest when NSE cut-off level was 12.5 ng/ml. In Asia, by contrast, the lowest sensitivity (0.527, 95%CI: 0.345-0.701) and highest specificity (0.938, 95%CI: 0.750-0.987) were obtained when the NSE cut-off level was 20 ng/ml; the lowest specificity (0.861, 95%CI: 0.718-0.938) was obtained when cut-off level was 12.5 ng/ml; and the highest sensitivity (0.603, 95%CI: 0.488-0.707) was obtained when cut-off level was 10 ng/ml.

**Table 4:** The sensitivity and specificity of different cut-off levels

Cut-off (ng/ml)	No. of trials	No. of patients	Sensitivity	Specificity
**All**	35	9546	0.688(95%CI: 0.627- 0.743)	0.922(95%CI: 0.890- 0.944)
10	29	8323	0.688(95%CI: 0.621- 0.748)	0.918(95%CI: 0.879- 0.946)
12.5	24	7208	0.684(95%CI: 0.605- 0.754)	0.919(95%CI: 0.689- 0.951)
15	20	6098	0.668(95%CI: 0.570- 0.753)	0.933(95%CI: 0.880- 0.963)
20	12	4379	0.663(95%CI: 0.498- 0.797)	0.958(95%CI: 0.897- 0.983)
25	6	2750	0.733(95%CI: 0.416- 0.914)	0.986(95%CI: 0.943- 0.997)
**Europe**				
10	18	6420	0.730(95%CI: 0.661- 0.790)	0.933(95%CI: 0.899- 0.956)
12.5	15	5681	0.723(95%CI: 0.636- 0.796)	0.940(95%CI: 0.900- 0.964)
15	12	4745	0.723(95%CI: 0.604- 0.817)	0.953(95%CI: 0.912- 0.975)
20	7	3607	0.745(95%CI: 0.511- 0.891)	0.969(95%CI: 0.908- 0.990)
25	5	2442	0.803(95%CI: 0.460-0.951)	0.983(95%CI: 0.924- 0.996)
**Asia**				
10	11	1903	0.603(95%CI: 0.488- 0.707)	0.886(95%CI: 0.772- 0.947)
12.5	9	1527	0.594(95%CI: 0.474- 0.704)	0.861(95%CI: 0.718- 0.938)
15	8	1353	0.564(95%CI: 0.442- 0.678)	0.881(95%CI: 0.731- 0.953)
20	5	772	0.527(95%CI: 0.345- 0.701)	0.938(95%CI: 0.750- 0.987)
**ELISA**				
10	12	3098	0.759(95%CI: 0.653- 0.840)	0.901(95%CI: 0.831- 0.944)
12.5	11	2883	0.765(95%CI: 0.644- 0.855)	0.895(95%CI: 0.816- 0.942)
15	9	2465	0.769(95%CI: 0.603- 0.879)	0.912(95%CI: 0.837- 0.955)
20	6	1785	0.783(95%CI: 0.484- 0.933)	0.925(95%CI: 0.788- 0.976)
25	3	1455	0.754(95%CI: 0.703- 0.800)	0.928(95%CI: 0.911- 0.942)
**RIA**				
10	13	3641	0.628(95%CI: 0.521- 0.725)	0.953(95%CI: 0.906- 0.977)
12.5	9	2741	0.592(95%CI: 0.459- 0.712)	0.966(95%CI: 0.907- 0.988)
15	7	2049	0.542(95%CI: 0.390- 0.687)	0.978(95%CI: 0.925- 0.994)
20	5	1591	0.504(95%CI: 0.311- 0.696)	0.988(95%CI: 0.941- 0.998)
25	3	1295	0.438(95%CI: 0.395- 0.482)	0.992(95%CI: 0.983- 0.997)
**ECISA**				
10	4	1584	0.700(95%CI: 0.643- 0.753)	0.847(95%CI: 0.826- 0.866)
12.5	4	1584	0.700(95%CI: 0.643- 0.753)	0.847(95%CI: 0.826- 0.866)
15	4	1584	0.700(95%CI: 0.643- 0.753)	0.847(95%CI: 0.826- 0.866)

Two studies did not report the cut-off positive value of NSE; the cut-off value greater than or equal to 25 ng/ml were only found in one study, so this subgroup analysis could not conducted in Asian; ELISA: enzyme linked immunosorbent assay; ECSIA: electro- chemiluminescence immunoassay; RIA= radioimmunoassay assay. As for ECISA, when cut-off value was 20 ng/ml, only one studies was involved.

No single NSE cut-off level achieved both the highest sensitivity and specificity for ELISA, RIA or ECISA. When NSE cut-off level was 25 ng/ml, ELISA had the highest specificity, and when cut-off level was 20 ng/ml, the sensitivity was highest. For RIA, the highest sensitivity was obtained at 10 ng/ml, and the highest specificity was obtained at 25 ng/ml. For ECSIA, when the cut-off level was 10 ng/ml, 12.5 ng/ml or 15 ng/ml, the sensitivity was 0.700 (95%CI: 0.643-0.753) and specificity was 0.847 (95%CI: 0.826-0.866) across 4 trials. However when cut-off value was 20 ng/ml, only one study was involved.

### Publication bias

**Figure 10:**
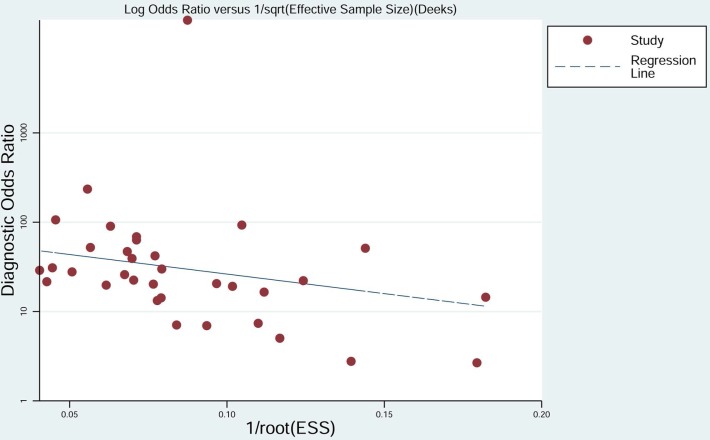
Deek's funnel plot evaluating publication bias in the included studies

To assess the publication bias for the diagnostic tests, we used Deek's funnel plots of lnDOR against 1/ESS1/2 or, equivalently, against (1/n1 + 1/n2)1/2, which is proportional to 1/ESS1/2 [42]. The *p* value obtained from the funnel plot was 0.001, indicating the presence of publication bias in this meta-analysis (Figure 10).

## DISCUSSION

NSE, a traditional tumor biomarker, has been well studied over the years [43–45], and it is commonly used in the diagnosis of SCLC. Although NSE cannot replace histological results, it can be particularly helpful in cases where it is not possible to establish a final diagnosis through biopsy. But to precisely determine the diagnostic efficacy of NSE levels, they should be subjected to pool analysis, and the precise impact of the tumor site and detection method must be determined. Moreover, the most suitable NSE cut-off level should also be established. Our study addressed these issues to a degree.

This systematic review indicated that NSE levels are highly useful for detecting SCLC in patients with benign lung diseases and in healthy individuals. NSE showed high specificity with lower sensitivity. However, the diagnostic performance was much better in Europe than in Asia. The diagnostic performance also differed depending on whether ELISA, RIA and ECISA were used to screen for SCLC. For all countries, ELISA had the highest sensitivity, while RIA had the highest specificity. Likewise, when considered separately in Europe and Asia, the highest sensitivity and specificity were obtained with ELISA and RIA, respectively, when using NSE levels in the diagnosis of SCLC.

There is currently doubt about the appropriate cut-off level for NSE. Normal levels of NSE are less than 12.5 ng/ml. Nonetheless, when cut-off levels were 10 ng/ml or 12.5 ng/ml, the sensitivity and specificity were similar to those obtained in some studies with higher cut-off levels. The best diagnostic performance was obtained with a NSE cut-off level of 25 ng/ml, while the lowest diagnostic performance was obtained at a cut-off level of 12.5 ng/ml. Consistent with all studies, in Europe, the highest and lowest diagnostic performances were obtained at 25 ng/ml and 12.5 ng/ml, respectively. In Asia, however, the highest sensitivity and specificity were obtained at 10 ng/ml and 20 ng/ml, and the lowest sensitivity and specificity were at 20 ng/ml and 12.5 ng/ml. In Europe, therefore, 25 ng/ml may be the most suitable cut-off level. In Asia, however, no single cut-off value had highest sensitivity and specificity, suggesting more studies are warranted.

Our meta-analysis included 33 studies with 9546 samples obtained through a comprehensive search strategy. Meta-regression and subgroup analyses for different regions, detection methods, cut-off levels, and sample sizes were conducted to investigate sources of heterogeneity. Nonetheless, our review has several limitations. First, only papers in English or Chinese were included in our review, so studies in other languages may have been excluded. Second, significant publication bias exists in this review, which may reduce the power of our analysis. Finally, some studies were rated “high risk”, and the item flow and timing may have impacted the pooled effects. In clinical practice, because it is difficult to completely fit flow and timing while guaranteeing a sufficient sample size, there is eventually an inappropriate interval between the index test and reference standard.

In sum, our analysis indicates that NSE levels provide high diagnosis accuracy for early detection of SCLC in patients with benign lung diseases and healthy individuals, though the diagnostic performance is better in Europe than in Asia. ELISA had the highest sensitivity and RIA had the highest specificity. In the clinic, NSE should be considered together with the clinical symptoms, image results and histopathology.

## MATERIALS AND METHODS

This is not primary research; no ethical approval or informed consent was necessary for this meta-analysis. Our review was conducted according to the guidelines of the *Cochrane Handbook for Diagnostic Test Accuracy Reviews*, available at http://srdta.cochrane.org. The protocol is registered with the Centre for Reviews and Dissemination PROSPERO database (available at: https://www.crd.york.ac.uk/PROSPERO/display_record.asp?ID=CRD42014010777).

### Search strategy

A comprehensive search of the PubMed, EMBASE, Web of Science, Cochrane library, and Chinese biomedical literature databases was conducted to identify studies published through September 2016. Search terms included neuron-specific enolase and small cell lung cancer. Papers published in English and Chinese were included in our review. Reference lists of the reports selected in the original search were also examined. The strategy used for PubMed is summarized in Supplementary Data 1.

### Study inclusion and exclusion criteria

Titles, abstracts, and full texts were independently screened by two reviewers, and a third reviewer acted to resolve any disagreements. Studies included in our review met the following criteria: 1) NSE was used to detect SCLC in patients with benign lung diseases and healthy individuals; 2) data such as true positive (TP), false positive (FP), false negative (FN), and true negative (TN) were available in the studies; 3) diagnostic tests was designed in the studies. Excluded were the following: 1) reviews and meeting abstracts; 2) papers from which the extracted data was not sufficient; 3) case reports.

### Data extraction and quality assessment

Study features (last name of the first author, year of publication, and country), number of samples and outcome data (TP, FP, FN, and TN) were extracted by two reviewers. The methodological quality of the included studies was assessed using the QUADAS-2 tool and Review Manager 5.3 (The Nordic Cochrane Centre, The Cochrane Collaboration, 2014). With respect to the Cochrane guidelines, we assigned low, high, or unclear risk of bias values to the patient selection; index tests, reference standards, and item flow and timing domains were also evaluated. Applicability concerns were evaluated in the first three domains.

### Statistical analysis

Pooled sensitivity, specificity, PLR, NLR, DOR, and AUC and associated 95% confidence intervals (CIs) were calculated using a bivariate regression model. Heterogeneity was assessed using a bivariate boxplot, Chi-square test, and inconsistency index (*I*^2^). If *I*^2^ was greater than 50%, significant heterogeneity would be considered to exist in the studies. In addition, meta-regression and subgroup analyses were used to investigate potential sources of heterogeneity. A likelihood ratio scattergram was used to evaluate the exclusion and confirmation capacities of the index test. Finally, clinical utility and publication bias were assessed using a Fagan diagram and Deek's plot. The statistical analysis was conducted using STATA version 12.0 (Stata Crop, college Station, TX).

## SUPPLEMENTARY DATA



## Abbreviations

NSE: serum neuron-specific enolase
SCLC: small cell lung cancer
QUADAS-2: Quality Assessment of Diagnostic Accuracy Studies
ELISA: enzyme linked immunosorbent assays
ECSIA: electro-chemiluminescence immunoassays
RIA: radioimmunoassay assays
SROC: summary receiver operator characteristic
PLR: positive likelihood ratio
NLR: negative likelihood ratio
DOR: diagnostic odds ratio
AUC: area under the curve
TP: true positive
FP: false positive
FN: false negative
TN: true negative
