# Supplementary material for: Systematic review and meta-analysis of the efficacy of serum neuron-specific enolase for early small cell lung cancer screening
Source: Oncotarget. 2017 May 11;8(38):64358–72. doi: 10.18632/oncotarget.17825 (PMC5610008; doi:10.18632/oncotarget.17825)
Supplement: Supplementary file 1 [file oncotarget-08-64358-s001.pdf]

Systematic review and meta-analysis of the efficacy of serum neuron-specific enolase for early small cell lung cancer screening

SUPPLEMENTARY DATA

Supplementary Data 1: The Search Strategy for PubMed

#1 Search ((("Small Cell Lung Carcinoma"[Mesh]) OR (Small Cell Lung Cancer[Title/Abstract] OR Oat Cell Lung Cancer[Title/Abstract] OR Small Cell Cancer Of The Lung[Title/Abstract] OR Carcinoma, Small Cell Lung[Title/Abstract] OR Oat Cell Carcinoma of Lung[Title/Abstract]))).

#2 Search ((NSE[Title/Abstract] OR Nervous System-Specific Enolase[Title/Abstract] OR Enolase, Nervous System-Specific[Title/Abstract] OR Nervous System Specific Enolase[Title/Abstract] OR System-Specific Enolase, Nervous[Title/Abstract] OR Neuron-Specific Enolase[Title/Abstract] OR Enolase, Neuron-Specific[Title/Abstract] OR Neuron Specific Enolase[Title/Abstract])).

#3 #1 AND #2.

Supplementary Data 2: The meta-regression of sensitivity, specificity and joint model for methodological quality

**Sensitivity**					
**Parameter**		**Estimate(95%CI)**	**Coef**	**Z**	***P*>|z|**
Risk of bias	patient selection	0.73 [0.59 - 0.83]	0.97	0.64	0.52
	flow and timing domains	0.74 [0.68 - 0.80]	1.06	2.79	0.01*
Applicability concerns	patient selection	0.73 [0.65 - 0.79]	0.97	1.45	0.15
	index test	0.76 [0.67 - 0.83]	1.16	2.00	0.05
	reference standard	0.68 [0.61 - 0.73]	0.73	-1.12	0.26
**Specificity**					
**Parameter**		**Estimate(95%CI)**	**Coef**	**Z**	***P*>|z|**
Risk of bias	patient selection	0.92 [0.84 - 0.96]	2.44	-0.05	0.96
	flow and timing domains	0.90 [0.86 - 0.94]	2.25	-1.48	0.14
Applicability concerns	patient selection	0.91 [0.86 - 0.94]	2.34	-0.73	0.46
	index test	0.92 [0.86 - 0.96]	2.47	0.02	0.99
	reference standard	0.92 [0.89 - 0.95]	2.48	0.24	0.81
**Joint model**					
**Parameter**		**I-squared(95%CI)**	**LRTChi**	***P* Value**	
Risk of bias	patient selection	0.00 [0.00 - 100.00]	0.44	0.80	
	flow and timing domains	74.00 [42.66 - 100.00]	7.69	0.02*	
Applicability concerns	patient selection	8.53 [0.00 - 100.00]	2.19	0.34	
	index test	53.71 [0.00 - 100.00]	4.32	0.12	
	reference standard	0.00 [0.00 - 100.00]	1.23	0.54	

* P<0.05.

Some eligible studies were rated to be as high risk of bias, and may overestimate or underestimate the pooled effects. As for *the chapter 9 of Cochrane Handbook for Diagnostic Test Accuracy Reviews* (http://methods.cochrane.org/sites/methods.cochrane.org.sdt/files/public/uploads/ch09_Oct09.pdf), we explored the impact of poor methodological quality using meta-regression analyses. The meta-analysis model is extended by including the quality assessment item as a covariate. We considered each item as a binary indicator, and combined the 'unclear' judgments with the 'no' judgments for all items of applicability concerns and risk of bias. Because of index test and reference standard were all be assigned as unclear or high risk, and meta-regression could not been conducted. The results indicated that only flow and timing domain for risk of bias may had impact on sensitivity and joint model, while others did not. When flow and timing was assigned as "unclear" or "high risk", the sensitivity was 0.605 (95%CI: 0.500-0.700) and specificity was 0.949 (95%CI: 0.893-0.977), and the sensitivity was 0.737 (95%CI: 0.667-0.796) and specificity was 0.904 (95%CI: 0.866-0.932) when assigned "low risk".
